# Comparative Effects of Sertraline, Methotrexate, and Biologic Therapies on Circulating Inflammatory and Neurotrophic Mediators and Striatal Gene Expression in Collagen-Induced Arthritis

**DOI:** 10.3390/ijms27167471

**Published:** 2026-08-21

**Authors:** Grzegorz Chmielewski, Mateusz Mikiewicz, Jakub Kuna, Łukasz Jaśkiewicz, Joanna Czerwińska, Małgorzata Wróbel, Edyta Kaczorek-Łukowska, Michał S. Majewski, Magdalena Krajewska-Włodarczyk

**Affiliations:** 1 Department of Rheumatology, School of Medicine, Collegium Medicum, University of Warmia and Mazury in Olsztyn, 10-719 Olsztyn, Poland; kuna.jakub@wp.pl; 2 Department of Pathological Anatomy, Faculty of Veterinary Medicine, University of Warmia and Mazury in Olsztyn, 10-719 Olsztyn, Poland; mateusz.mikiewicz@uwm.edu.pl; 3 Department of Human Physiology and Pathophysiology, School of Medicine, Collegium Medicum, University of Warmia and Mazury in Olsztyn, 10-719 Olsztyn, Poland; lukasz.jaskiewicz@uwm.edu.pl; 4 Department of Dermatology, Sexually Transmitted Diseases and Clinical Immunology, School of Medicine, Collegium Medicum, University of Warmia and Mazury in Olsztyn, 10-719 Olsztyn, Poland; joanna.czerwinska@uwm.edu.pl; 5 Department of Microbiology and Clinical Immunology, Faculty of Veterinary Medicine, University of Warmia and Mazury in Olsztyn, 10-719 Olsztyn, Poland; malgorzata.wrobel@uwm.edu.pl (M.W.); edyta.kaczorek@uwm.edu.pl (E.K.-Ł.); 6 Department of Pharmacology and Toxicology, Faculty of Medicine, University of Warmia and Mazury in Olsztyn, 10-719 Olsztyn, Poland; michal.majewski@uwm.edu.pl

**Keywords:** rheumatoid arthritis, collagen-induced arthritis, sertraline, inflammation, myokines

## Abstract

Rheumatoid arthritis is often accompanied by neuropsychiatric manifestations, suggesting interactions between systemic inflammation and the central nervous system. This study evaluated the effects of sertraline, compared with methotrexate, infliximab, and tocilizumab, on circulating inflammatory and neurotrophic mediators and the striatal expression of selected CNS-related genes in collagen-induced arthritis. Male Wistar rats were assigned to seven groups: adjuvant control, untreated arthritis, methotrexate, sertraline, methotrexate plus sertraline, infliximab, or tocilizumab. At week 12, circulating IL-6, IL-15, IL-10, BDNF, myostatin, and irisin were measured, and striatal BDNF, IL-1β, and GFAP expression was assessed by quantitative real-time PCR. IL-6 concentrations differed between groups, with the lowest values after infliximab treatment and the highest in untreated arthritic rats and animals receiving methotrexate plus sertraline or tocilizumab. IL-15 was highest in untreated arthritis, whereas IL-10 was generally higher in treated groups and peaked after tocilizumab. The IL-6/IL-10 ratio was lower in all treated groups than in untreated arthritis, with the most pronounced reduction observed after infliximab. Circulating BDNF concentrations were highest in the infliximab- and tocilizumab-treated groups. Striatal BDNF expression differed significantly between groups, whereas no significant between-group differences were detected in striatal GFAP or IL-1β expression. No significant between-group differences were observed for circulating myostatin or irisin. Sertraline-containing regimens were associated with lower IL-6/IL-10 ratios than untreated arthritis, whereas infliximab and tocilizumab were associated with the most pronounced changes in systemic inflammatory and neurotrophic parameters.

## 1. Introduction

Rheumatoid arthritis (RA) is a chronic autoimmune disorder characterized by persistent synovial inflammation and progressive destruction of articular cartilage and subchondral bone. Although the disease primarily affects joints, it is increasingly recognized as a systemic condition [1]. Circulating inflammatory mediators released during active disease contribute not only to joint pathology but also to extra-articular manifestations, reflecting the complex interplay between peripheral immune activation and systemic physiological responses [2]. Among the most frequent comorbidities observed in patients with RA are neuropsychiatric disorders, particularly depression. The coexistence of these conditions substantially worsens clinical outcomes by reducing quality of life, impairing adherence to therapy, and limiting the likelihood of achieving sustained remission [3,4,5,6]. Epidemiological studies indicate that depressive symptoms occur significantly more often in individuals with RA than in the general population [7]. This relationship appears to be bidirectional, as chronic inflammation, pain, and functional limitations associated with RA may promote the development of depressive symptoms, while depression itself may exacerbate inflammatory activity and worsen disease progression [8].

Increasing evidence indicates that inflammatory pathways play an important role in the biological mechanisms underlying depression. Elevated circulating levels of proinflammatory mediators, including interleukin-1β (IL-1β), interleukin-6 (IL-6), and tumour necrosis factor-α (TNF-α), have been reported in patients with depressive disorders [9]. Persistent immune activation may disrupt neuroendocrine regulation and influence neurotransmitter metabolism, particularly within serotonergic and glutamatergic systems. These alterations may contribute to neuroinflammatory processes in the brain, affecting neuronal plasticity and emotional regulation [9].

Communication between peripheral tissues and the central nervous system is mediated by a variety of signalling molecules, including muscle-derived cytokines and peptides known as myokines. These molecules participate in metabolic regulation, inflammatory signalling, and neurobiological processes [10,11]. Myokines such as IL-6, interleukin-15 (IL-15), irisin, and myostatin (MSTN) have been implicated in systemic inflammatory responses and may influence brain function through immune–neural interactions [11]. In parallel, neurotrophic factors such as brain-derived neurotrophic factor (BDNF) play a critical role in neuronal survival, synaptic plasticity, and mood regulation [12]. Alterations in BDNF expression, together with markers of glial activation such as glial fibrillary acidic protein (GFAP) and proinflammatory cytokines including IL-1β, are frequently associated with neuroinflammatory states and depressive-like behaviour [13].

Selective serotonin reuptake inhibitors (SSRIs), including sertraline, are widely used in the pharmacological treatment of depression. Their principal mechanism involves inhibition of serotonin reuptake in the central nervous system [14]. In addition to its neuropharmacological effects, sertraline may influence inflammatory signalling and immune-cell activity by reducing proinflammatory cytokine production and enhancing expression of anti-inflammatory mediators [15]. Such effects may indicate that SSRIs may influence interactions between systemic inflammation and neurobiological processes.

Despite the substantial therapeutic progress achieved through the introduction of disease-modifying antirheumatic drugs (DMARDs) and targeted biological therapies, effective long-term control of RA remains challenging for many patients. A considerable proportion of individuals do not achieve full clinical remission or experience incomplete responses to treatment [16,17,18]. These limitations underscore the importance of further research into systemic mechanisms connecting inflammatory processes with neurobiological pathways.

The striatum was selected as the brain region of interest because of its involvement in corticostriatal circuits regulating motivation, reward processing, and motor activity. These functions may be particularly sensitive to systemic inflammatory signalling. Previous studies have shown that inflammatory activation can affect basal ganglia and striatal function, including dopaminergic neurotransmission and reward-related neural responses, and these alterations have been associated with reduced motivation, anhedonia, fatigue, and psychomotor slowing [19,20]. Accordingly, analysis of the striatum provided a targeted approach to investigate selected CNS-related molecular responses to systemic inflammation and pharmacological treatment. However, the striatum represents only one component of the broader neural circuitry involved in mood regulation.

The aim of the present study was to evaluate circulating levels of selected inflammatory, neurotrophic, and muscle-related mediators, including IL-6, IL-15, IL-10, BDNF, myostatin (MSTN), and irisin, in a rat model of collagen-induced arthritis, together with striatal expression of selected CNS-related genes, including BDNF, IL-1β, and GFAP. The effects of sertraline were compared with methotrexate, infliximab, and tocilizumab to explore potential associations between systemic inflammatory regulation and molecular responses in the striatum.

## 2. Results

Systemic levels of inflammatory mediators and myokines showed distinct, analyte-specific patterns across the experimental groups (Table 1).

**Figure 1:**
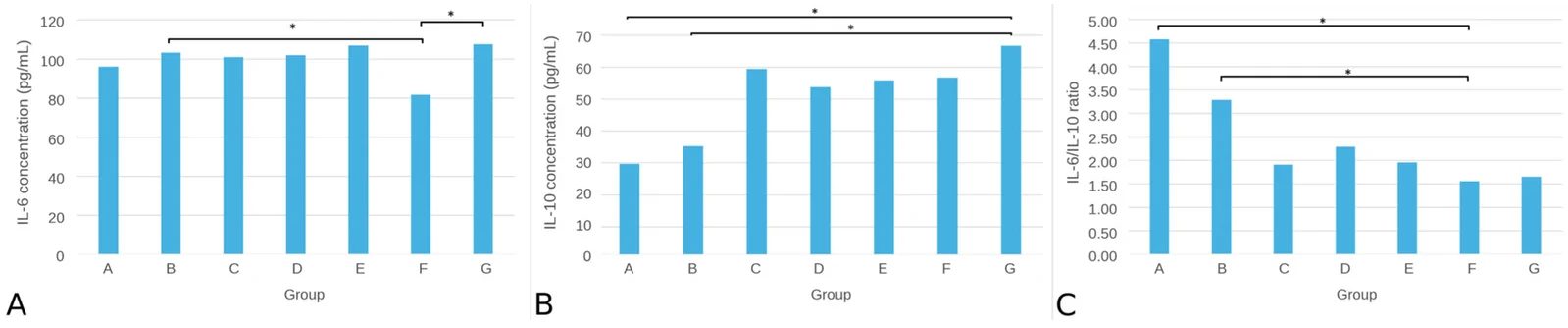
Serum concentrations of IL-6 and IL-10 and the IL-6/IL-10 ratio across the experimental groups. (**A**) Mean serum IL-6 concentrations. Group F showed significantly lower IL-6 concentrations than groups B (*p* = 0.016) and G (*p* = 0.003). (**B**) Mean serum IL-10 concentrations. Group G showed significantly higher IL-10 concentrations than groups A (*p* = 0.016) and B (*p* = 0.038). (**C**) IL-6/IL-10 ratio. Group F exhibited a significantly lower IL-6/IL-10 ratio than groups A (*p* = 0.032) and B (*p* = 0.037), * *p* < 0.05.

IL-6 concentrations differed significantly between groups (H(6) = 18.13, *p* = 0.006, η^2^H = 0.248). The highest levels were observed in groups G, E, and B, whereas groups C and D displayed comparable intermediate values. Lower concentrations were noted in group A and, most prominently, in group F. Pairwise comparisons confirmed significantly reduced IL-6 levels in group F compared to groups B (*p* = 0.016) and G (*p* = 0.003) (Figure 1A).

IL-10 concentrations differed significantly between groups (H(6) = 19.62, *p* = 0.003, η^2^H = 0.278), indicating a shift toward an anti-inflammatory profile. Groups A and B were characterized by relatively low baseline levels, whereas groups C–F showed uniformly elevated concentrations, with the highest levels observed in group G. This increase reached statistical significance when comparing group G with both group A (*p* = 0.016) and group B (*p* = 0.038) (Figure 1B).

These changes were mirrored in the IL-6/IL-10 ratio (H(6) = 18.59, *p* = 0.005, η^2^H = 0.257), which was highest in groups A and B, reflecting a predominance of pro-inflammatory signalling. In contrast, all remaining groups (C–G) exhibited lower ratios, consistent with a shift toward anti-inflammatory balance. This reduction was statistically significant in group F compared to both group A (*p* = 0.032) and group B (*p* = 0.037), while other comparisons did not reach significance despite a consistent downward trend (Figure 1C).

**Figure 2:**
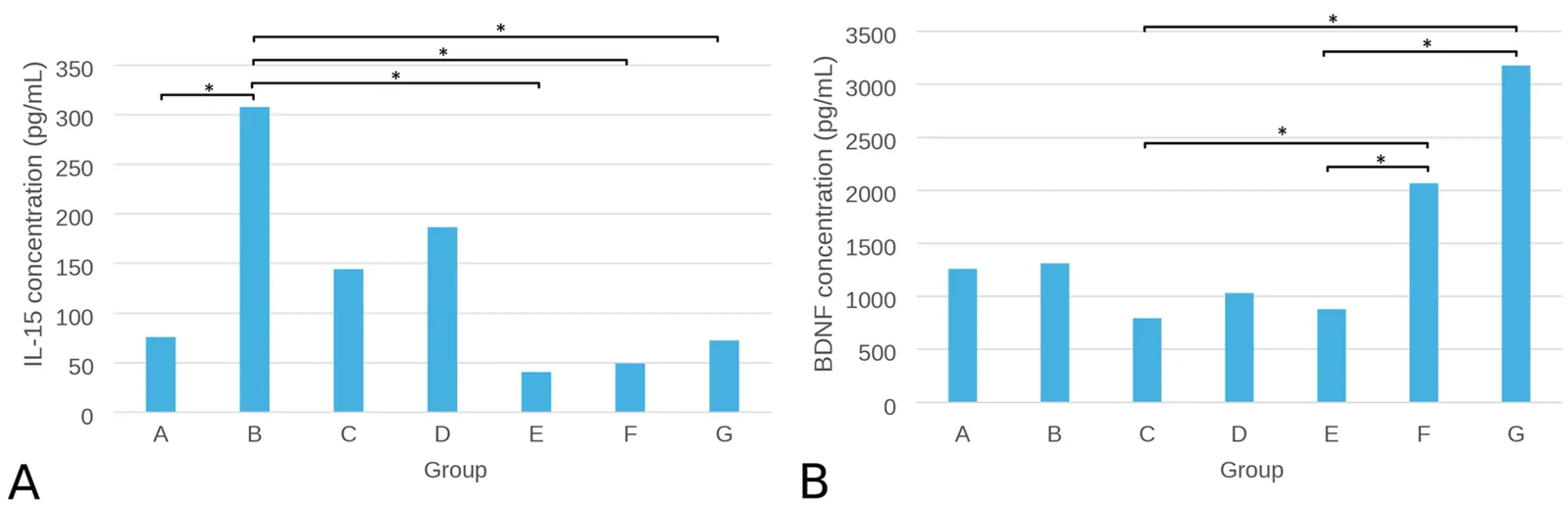
Serum concentrations of IL-15 and BDNF across experimental groups. (**A**) Mean serum IL-15 concentrations. Group B showed significantly higher IL-15 concentrations than groups A (*p* = 0.019), E (*p* < 0.001), F (*p* = 0.001), and G (*p* = 0.02). (**B**) Mean serum BDNF concentrations. Group G showed significantly higher BDNF concentrations than groups C (*p* < 0.001), D (*p* = 0.011), and E (*p* < 0.001). Additionally, group F showed significantly higher BDNF concentrations than groups C (*p* = 0.007) and E (*p* = 0.02), * *p* < 0.05.

IL-15 concentrations differed significantly between groups (H(6) = 29.77, *p* < 0.001, η^2^H = 0.485), with a clear peak in group B, exceeding all other groups. Moderate levels were observed in groups D and C, whereas the remaining groups exhibited substantially lower concentrations. Accordingly, IL-15 levels in group B were significantly higher than in groups A (*p* = 0.019), E (*p* < 0.001), F (*p* = 0.001), and G (*p* = 0.02) (Figure 2A).

Circulating BDNF concentrations differed significantly between groups (H(6) = 34.59, *p* < 0.001, η^2^H = 0.583), with the highest concentrations observed in groups G and F. Intermediate values were recorded in groups A, B, and D, whereas the lowest levels were found in groups C and E. Pairwise comparisons showed that group G had significantly higher BDNF levels than groups C (*p* < 0.001), D (*p* = 0.011), and E (*p* < 0.001). Similarly, group F exhibited higher levels compared to groups C (*p* = 0.007) and E (*p* = 0.02) (Figure 2B).

In contrast, no significant between-group differences were detected for MSTN (H(6) = 9.51, *p* = 0.147, η^2^H = 0.072) or irisin (H(6) = 9.47, *p* = 0.149, η^2^H = 0.071). Although some variability in group-level values was observed, these differences did not reach statistical significance.

### Striatal Gene Expression

At the transcriptional level, striatal BDNF expression differed significantly between groups in the primary ΔCt-based analysis (*p* = 0.024). Pairwise comparisons indicated significant differences between groups A and C (*p* = 0.019) and A and D (*p* = 0.030), whereas no other comparisons reached statistical significance. Relative fold-change values are presented descriptively to illustrate the magnitude and direction of BDNF expression differences.

No significant between-group differences in striatal GFAP expression were detected in the primary ΔCt-based analysis (*p* = 0.441). Fold-change values are presented descriptively and were not subjected to a separate inferential statistical analysis.

No significant between-group differences in striatal IL-1β expression were detected in the primary ΔCt-based analysis (*p* = 0.217). Fold-change values are presented descriptively and were not subjected to a separate inferential statistical analysis.

## 3. Discussion

The present findings suggest that the applied therapeutic interventions may differentially modulate inflammatory cytokine profiles and, to a lesser extent, myokine responses in the collagen-induced arthritis (CIA) model, potentially reflecting distinct mechanisms of action across treatment groups. The most pronounced anti-inflammatory effects were observed in animals treated with biological agents targeting key cytokine pathways.

IL-6 concentrations were higher in untreated CIA rats than in the adjuvant control group, confirming its role as a marker of systemic inflammation in collagen-induced arthritis [21]. Among the treated groups, infliximab was associated with the lowest IL-6 levels, which were significantly reduced compared with both untreated CIA rats and tocilizumab-treated animals. This finding may reflect attenuation of downstream IL-6-related signalling following TNF-α blockade, in line with the upstream role of TNF-α in the inflammatory cascade and with clinical reports of reduced serum IL-6 concentrations during TNF inhibitor therapy [22,23]. Nevertheless, circulating IL-6 represents only one component of the inflammatory response, and the present results do not establish a direct causal relationship between TNF-α inhibition and the observed reduction in IL-6.

In contrast, methotrexate- and sertraline-treated rats, as well as animals receiving combined treatment, showed intermediate IL-6 concentrations, with no statistically significant reduction relative to untreated CIA rats. The absence of a significant reduction in circulating IL-6 should not necessarily be interpreted as evidence of insufficient anti-inflammatory activity. Previous studies have shown that methotrexate may reduce arthritis severity and anti-collagen type II antibody levels without consistently suppressing macrophage-derived IL-6 production, suggesting that its therapeutic effects may involve mechanisms not directly reflected by this circulating cytokine [24]. At the same time, the effect of methotrexate on IL-6 appears to be variable and dependent on disease stage, treatment duration, and the timing of assessment. Reductions in IL-6 have been reported during both early and longer-term treatment, whereas in other studies methotrexate primarily affected mediators such as sIL-2R and soluble TNF receptors, with less consistent changes in IL-6, TNF-α, or IL-1β [25,26,27,28,29].

Taken together, these findings suggest that while methotrexate can modulate IL-6, its effects are context-dependent and may not be consistently reflected in systemic cytokine levels at a single time point. This may also reflect biological variability, timing of sampling, or differences between systemic cytokine concentrations and local joint inflammation.

The elevated IL-6 concentration observed in the tocilizumab-treated group should be interpreted with caution. Tocilizumab blocks IL-6 receptor signalling rather than IL-6 production; therefore, increased circulating IL-6 may reflect reduced receptor-mediated clearance instead of persistent inflammatory activity. This phenomenon has been consistently reported in previous studies, where tocilizumab treatment resulted in a concentration-dependent increase in circulating IL-6 levels, reflecting reduced receptor-mediated clearance rather than enhanced cytokine production [30]. Similarly, administration of anti-IL-6 receptor antibodies has been shown to increase circulating IL-6 concentrations without necessarily stimulating additional IL-6 synthesis, further supporting impaired receptor-mediated clearance as a plausible explanation [31].

IL-15 showed a distinct distribution pattern from IL-6, with the highest concentration observed in the untreated CIA group. Its significant elevation relative to adjuvant control supports involvement in the systemic inflammatory response associated with collagen-induced arthritis [32]. This interpretation is supported by previous experimental and clinical studies indicating that IL-15 contributes to autoimmune arthritis by promoting T-cell activation, sustaining T-cell-macrophage interactions, and amplifying downstream proinflammatory cytokine responses [33]. Nevertheless, the present data demonstrate an association rather than a direct causal role for IL-15 in disease progression.

In the present study, untreated CIA rats showed significantly higher IL-15 concentrations than animals treated with methotrexate plus sertraline, infliximab, or tocilizumab. This pattern is consistent with the possibility that these therapeutic regimens attenuated IL-15-associated inflammatory activity. The reduced IL-15 level in the combination treatment may indicate a stronger modulation of this pathway than that achieved by either methotrexate or sertraline alone. This interpretation is in line with previous findings indicating that methotrexate may interfere with IL-15-mediated inflammatory responses, including T-cell TNF production and fibroblast–T-cell interactions in rheumatoid arthritis models [34]. However, as direct comparisons between the monotherapy groups and the methotrexate plus sertraline group remained non-significant, this interpretation should be considered hypothesis-generating rather than conclusive.

The low IL-15 levels observed after infliximab treatment may reflect suppression of the broader cytokine network. In tocilizumab-treated rats, reduced IL-15 concentrations may suggest that IL-6 receptor blockade indirectly limited inflammatory cell activation and cytokine amplification [35,36]. Nevertheless, the present study did not directly assess the mechanisms linking TNF-α or IL-6 receptor blockade to IL-15 regulation, and these explanations therefore remain speculative. In contrast, IL-15 concentrations in methotrexate- and sertraline-treated rats were moderate and remained comparable to those observed in untreated CIA rats. Under the present experimental conditions, this finding may indicate that either monotherapy alone did not produce a sufficiently pronounced change in circulating IL-15 to be detected at the terminal time point. It does not, however, exclude effects on local IL-15 signalling within affected tissues or transient changes occurring earlier during treatment.

IL-10 concentrations were relatively low in adjuvant controls and untreated CIA rats, whereas higher levels were observed across all treated groups. Tocilizumab-treated rats showed the highest IL-10 concentrations, which were significantly higher than those observed in adjuvant controls and untreated CIA rats. This may indicate activation of anti-inflammatory or regulatory mechanisms following IL-6 receptor blockade. Previous experimental studies support the protective role of IL-10 in collagen-induced arthritis, suggesting that its effects are mediated mainly through regulation of inflammatory effector mechanisms rather than direct suppression of the anti-collagen antibody response [37,38,39].

A similar shift was reflected in the IL-6/IL-10 ratio. The highest ratios were observed in adjuvant controls and untreated CIA rats; however, in adjuvant controls this likely resulted mainly from low IL-10 levels rather than CIA-related inflammatory activity. All treated groups showed lower IL-6/IL-10 ratios, suggesting a shift toward a more anti-inflammatory cytokine profile [38,40]. This reduction was statistically significant only after infliximab treatment, possibly reflecting the combination of reduced IL-6 and relatively increased IL-10 following TNF-α blockade [40]. Overall, the increased IL-10 concentration after tocilizumab treatment and the reduced IL-6/IL-10 ratio after infliximab treatment indicate treatment-associated shifts toward a more anti-inflammatory systemic cytokine profile. These findings suggest differential effects of IL-6 receptor and TNF-α blockade on systemic cytokine balance.

A notable finding of the present study was the increase in circulating BDNF levels after biological treatment, particularly following tocilizumab and infliximab administration. BDNF is a neurotrophic factor involved in neuronal survival, synaptic plasticity, and neurogenesis, and its circulating concentrations may be influenced by inflammatory status [41]. Previous experimental studies have reported associations between inhibition of TNF-α or IL-6 signalling and changes in BDNF expression or neurobehavioral outcomes in inflammatory and stress-related models [42,43,44]. In the present study, the coexistence of higher circulating BDNF concentrations with changes in the systemic cytokine profile may therefore indicate an association between inflammatory regulation and peripheral BDNF levels. However, circulating BDNF cannot be considered a direct measure of BDNF activity within the CNS, particularly because peripheral BDNF concentrations may also be influenced by extracerebral sources and platelet-related mechanisms [45]. Moreover, striatal BDNF expression did not show consistent upregulation following biological treatment. Therefore, the present findings should not be interpreted as direct evidence of central neuroprotection, but rather as treatment-associated differences in circulating BDNF that warrant further investigation at the tissue and functional levels.

In contrast to the changes observed in cytokines and BDNF, MSTN levels remained stable across all groups, indicating that the applied interventions did not significantly affect systemic pathways associated with muscle mass regulation [46]. Previous experimental work has shown that CIA may be associated with reduced mobility, body weight loss, and skeletal muscle wasting, involving activation of muscle proteolytic and regenerative pathways [47]. Myostatin signalling was not detectably modulated by anti-inflammatory treatment in this CIA model. Alternatively, the duration of treatment or the systemic nature of the measurement may have been insufficient to reveal local changes in muscle-related signalling.

Similarly, irisin levels remained comparable across groups, despite some variability. Given that irisin concentrations may be influenced by multiple metabolic, physiological, and activity-related factors [48], the absence of consistent differences suggests that neither CIA nor the applied pharmacological interventions were associated with consistent systemic changes in this myokine under the present experimental conditions.

No significant between-group differences in striatal GFAP expression were detected in the primary ΔCt-based analysis. Although GFAP is commonly used as an astrocyte-associated marker, changes in GFAP mRNA expression alone cannot be considered direct evidence of astrocyte activation or neuroinflammation [49,50]. Previous studies have demonstrated region-specific alterations in brain molecular markers, including GFAP, in experimental collagen-induced arthritis. However, the absence of significant differences in the present study indicates that no treatment-related changes in striatal GFAP transcription were detected under the experimental conditions used. Confirmation at the protein and histological levels would be required to determine whether astrocyte-associated changes occur in this model. Similarly, IL-1β expression remained comparable across groups, indicating that treatment-related differences in this inflammatory transcript were not detected at the assessed time point. This does not exclude changes at the protein level or within specific cellular or tissue compartments, as IL-1β production and secretion are regulated at multiple post-transcriptional levels [51,52].

This study has several limitations. First, the collagen-induced arthritis model represents an experimentally induced form of inflammatory arthritis and may not fully reproduce the chronic, heterogeneous, and multifactorial course of human rheumatoid arthritis. Second, only male rats were included to reduce hormonal variability, which limits the generalizability of the findings to both sexes. Third, no validated tests specifically designed to assess depression- or anxiety-like behaviour were conducted; therefore, no direct conclusions regarding neuropsychiatric manifestations can be drawn from the present findings. Fourth, the CNS-related molecular analysis was restricted to mRNA expression of selected genes in the striatum, without protein-level confirmation or assessment of other brain regions relevant to mood regulation, such as the hippocampus and prefrontal cortex. Therefore, the findings should be regarded as region-specific and cannot be generalized to broader CNS or depression-related molecular responses. Finally, circulating and molecular markers were analysed only at a single terminal time point at week 12, which may not fully capture the temporal dynamics of arthritis progression or early treatment-related changes. Future studies should combine validated behavioural assessments with longitudinal analyses at both the transcript and protein levels to determine the functional significance of treatment-related neuroimmune changes in experimental arthritis.

## 4. Materials and Methods

### 4.1. Animals

The experiment was conducted using male Wistar rats (Cmdb:Wi) aged 8 weeks, with a body weight of 180 ± 20 g. Animals were obtained from the Centre for Experimental Medicine at the Medical University of Białystok (Białystok, Poland). After arrival, rats were kept under standardized laboratory conditions with controlled environmental parameters, including a temperature of 25 ± 1 °C, relative humidity of 40–60%, and a 12-h light/dark cycle. Throughout the experimental period, animals had unrestricted access to tap water and a standard laboratory diet.

Animals underwent a two-week acclimatization period and were handled regularly to reduce stress associated with human contact and experimental procedures. Environmental enrichment was provided in the form of shelters, tunnels, and nesting material to promote species-typical exploratory and nesting behaviour. Animals were regularly monitored for general health and signs of pain or distress. Humane endpoints were predefined before the experiment and included body weight loss exceeding 20%, persistent dehydration lasting more than 3 days, respiratory distress, prolonged immobility lasting more than 2 days, marked lethargy or apathy, and non-healing skin ulceration or infection. Welfare monitoring was performed twice daily and once daily on weekends and public holidays. No expected or unexpected adverse events were observed during the study.

All experimental procedures were performed in accordance with national and European regulations governing the use of animals in scientific research. The study protocol was approved by the Local Ethical Committee for Animal Experiments in Olsztyn (decision No. 19/2024, issued on 20 March 2024). The experiments complied with the Polish Act of 15 January 2015 on the Protection of Animals Used for Scientific or Educational Purposes (Journal of Laws 2015, item 266), implementing Directive 2010/63/EU of the European Parliament and of the Council on the protection of animals used for scientific purposes.

Only male rats were included in the study to limit biological variability associated with fluctuations in ovarian hormones. Hormonal status, particularly oestrogen levels, has been reported to influence both the susceptibility to and severity of collagen-induced arthritis in experimental models. Therefore, restricting the experiment to one sex enabled a more consistent evaluation of treatment-related effects in this arthritis model [53].

### 4.2. Induction of Collagen-Induced Arthritis

Following the two-week acclimatization period, all 56 male Wistar rats were assigned to seven groups (A–G; eight animals per group) prior to administration of collagen or vehicle. Group assignment was performed by simple randomization based on a computer-generated sequence. No stratification by body weight was applied, and stratification according to arthritis severity was not applicable because allocation was completed before arthritis induction. The allocation process was not concealed, and personnel conducting arthritis induction and administering the treatments were aware of the assigned groups. In contrast, all clinical evaluations were performed independently by two experienced investigators who were blinded to group allocation and treatment identity. No additional blinding was applied during sample collection and processing, ELISA and RT-qPCR measurements, or statistical analysis. No animals were excluded from the experiment. However, individual samples showing technically unreliable RT-qPCR results, characterized by markedly elevated Ct values for GAPDH, were excluded from the corresponding gene-expression analyses. These exclusions were applied consistently to both the primary ΔCt-based analysis and the descriptive fold-change calculations. No formal a priori sample-size calculation was performed. The group size of eight animals was selected based on group sizes used in previous experimental studies employing collagen-induced arthritis models and comparable pharmacological interventions, while also aiming to minimize the number of animals used in accordance with the principle of reduction. Experimental arthritis was induced using bovine type II collagen (Chondrex Inc., Woodinville, WA, USA) in accordance with the manufacturer’s instructions and previously described protocols [54,55,56,57].

### 4.3. Treatment

Group A served as the adjuvant-injected control and received vehicle consisting of 0.05 M acetic acid emulsified with incomplete Freund’s adjuvant without collagen administration and without pharmacological treatment. Group B represented the positive control group and consisted of rats with CIA that did not receive any therapeutic intervention. Group C included arthritic rats treated with methotrexate (Methofill; Accord Healthcare Poland, Pabianice, Poland) at a dose of 5 mg/kg administered intraperitoneally once weekly. Group D comprised animals with CIA receiving sertraline (Sertralina; KRKA, Nove mesto, Slovenia) at a dose of 10 mg/kg administered orally once daily. Group E consisted of rats treated with a combination of methotrexate (5 mg/kg, intraperitoneally, once weekly) and sertraline (10 mg/kg, orally, once daily). Group F included animals treated with infliximab (Remicade, Janssen-Cilag International NV, Beerse, Belgium) at a dose of 5 mg/kg administered intraperitoneally once weekly starting two weeks after arthritis induction. Group G consisted of rats treated with tocilizumab (RoActemra, Roche Pharma AG, Grenzach-Wyhlen, Germany) at a dose of 8 mg/kg administered intraperitoneally every two weeks beginning two weeks after arthritis induction.

Treatment regimens were selected based on pharmacologically active protocols reported in rodent models of inflammatory arthritis. Methotrexate was administered at 5 mg/kg intraperitoneally once weekly, and sertraline at 10 mg/kg orally once daily, consistent with previously used anti-inflammatory and neurobehavioral dosing schemes [58,59]. Infliximab and tocilizumab regimens were based on experimental studies targeting TNF-α and IL-6 receptor signalling, respectively [60,61].

Local anaesthesia with lidocaine was applied at the injection site during immunization. From the day of immunization, paracetamol was administered in the drinking water to provide analgesia.

### 4.4. Evaluation of Arthritis

To verify successful CIA induction, clinical monitoring of the animals was performed throughout the experiment. Hind-paw thickness was measured using Vernier callipers, and body weight was recorded at regular intervals (weeks 0, 4, 8, and 12; week 0 corresponded to the day of arthritis induction before the onset of clinical signs and prior to the initiation of treatment). In addition, the severity of arthritis was assessed using a standardized clinical scoring system based on the presence of redness and swelling of the hind limbs. Each hind paw was graded on a scale from 0 to 4 (0—no abnormalities; 1—mild redness and swelling limited to the tarsal region; 2—inflammatory changes extending toward the midfoot; 3—moderate swelling involving the metatarsal region; 4—pronounced swelling affecting the entire paw including the toes). The sum of scores from both hind paws constituted the arthritis index, with a maximum value of 8. These assessments were conducted primarily to verify the successful induction and progression of the CIA model and were not considered primary outcomes of the present study. Detailed longitudinal analyses of body weight, hind-paw thickness, arthritis severity, and related functional outcomes from the same experimental cohort have been reported separately and are therefore not reproduced here [62].

### 4.5. Euthanasia Procedure and Sample Collection

At the end of the experimental period (week 12), animals were deeply anesthetized by intraperitoneal administration of a ketamine–xylazine mixture consisting of ketamine (150 mg/kg; Vetketam, Vet-Agro, Lublin, Poland) and xylazine (20 mg/kg; Sedazin, Biowet, Puławy, Poland). After confirmation of surgical-depth anaesthesia, euthanasia was performed by cervical dislocation with transection of the spinal cord by an experienced operator. This method is permitted for laboratory rodents weighing less than 1 kg when preceded by appropriate anaesthesia, in accordance with Annex IV of Directive 2010/63/EU on the protection of animals used for scientific purposes. Immediately after euthanasia, blood samples were collected by cardiac puncture and centrifuged to obtain serum, which was used for the determination of selected circulating inflammatory, neurotrophic, and myokine-related factors. Brains were subsequently removed, and the striatum was carefully dissected on ice. Tissue samples were immediately frozen in liquid nitrogen and stored at −80 °C until molecular analysis.

### 4.6. Enzyme-Linked Immunosorbent Assay

Serum concentrations of IL-6 (pg/mL), IL-15 (pg/mL), IL-10 (pg/mL), BDNF (pg/mL), MSTN (pg/mL), and irisin (ng/mL) were determined using commercially available enzyme-linked immunosorbent assay (ELISA) kits: IL-6, IL-15, and IL-10, EIAab Science Co., Ltd. (Wuhan, China); BDNF, Invitrogen, Thermo Fisher Scientific (Waltham, MA, USA); MSTN, Wuhan Fine Biotech Co., Ltd. (FineTest; Wuhan, China); and irisin, Phoenix Pharmaceuticals, Inc. (Burlingame, CA, USA). The characteristics, suppliers, and catalogue numbers of the ELISA kits are presented in Table 2. Procedures were performed according to the manufacturer’s protocol. Absorbance values were measured at 450 nm using BioTek 800TS (Agilent Technologies, Inc., Santa Clara, CA, USA).

### 4.7. RNA Expression

Approximately 10 mg of frozen striatal tissue was used for total RNA isolation using TRIzol™ Reagent (Thermo Fisher Scientific, Waltham, MA, USA), according to the manufacturer’s instructions. RNA concentration and purity were assessed using a NanoDrop 2000 spectrophotometer (Thermo Fisher Scientific, Waltham, MA, USA). Only samples with A260/A280 ratios between 1.9 and 2.1 and A260/A230 ratios above 2.0 were included in further analyses. The isolated RNA was stored at −80 °C until further processing.

Residual genomic DNA was removed by treatment with DNase I (Invitrogen, Waltham, MA, USA). Complementary DNA (cDNA) was synthesized using a High-Capacity cDNA Reverse Transcription Kit (Life Technologies, Waltham, MA, USA), according to the manufacturer’s protocol. The amount of RNA used for cDNA synthesis was standardized and optimized to 0.5 µg per sample.

The expression levels of the genes encoding brain-derived neurotrophic factor (BDNF), interleukin-1 beta (IL-1β), and glial fibrillary acidic protein (GFAP) were determined by real-time quantitative PCR (RT-qPCR) using a LightCycler^®^ 96 Real-Time PCR System (Roche, Meylan, France) and DyNAmo HS SYBR Green PCR Master Mix (Life Technologies, Carlsbad, CA, USA). Each reaction was performed in a final volume of 20 µL and contained 10 µL of SYBR Green PCR Master Mix, 2 µL of cDNA, gene-specific amounts of forward and reverse primers, and nuclease-free water added to reach the final reaction volume. Primer sequences, primer amounts, and relevant references are provided in Table 3.

The amplification protocol consisted of an initial polymerase activation step at 95 °C for 15 min, followed by 40 cycles of denaturation at 94 °C for 10 s, primer annealing at 60 °C for 20 s, and elongation at 72 °C for 30 s. All reactions were performed in technical triplicates. Gene expression levels were normalized to the reference gene GAPDH for each individual sample by calculating ΔCt values (ΔCt = Ct_target − Ct_GAPDH). Samples excluded on technical grounds were omitted from the corresponding gene-expression analyses. Relative fold changes were subsequently calculated using the 2^−ΔΔCt^ method described by Livak and Schmittgen (2001) [67], with the control group serving as the calibrator, and were used for descriptive and graphical presentation of gene-expression changes.

### 4.8. Statistical Analysis

The normality of data distribution was assessed using the Shapiro–Wilk test. Differences between experimental groups in serum concentrations of IL-6, IL-15, IL-10, BDNF, MSTN, and irisin were analysed using the Kruskal–Wallis H test, followed by Dunn’s post-hoc test with Bonferroni correction for multiple comparisons. For Kruskal–Wallis analyses, ordinal eta-squared (η^2^H) was additionally calculated as an estimate of the overall effect size using the formula η^2^H = (H − k + 1)/(N − k), where H is the Kruskal–Wallis test statistic, k is the number of groups, and N is the total number of observations included in the analysis. For RT-qPCR data, between-group comparisons were performed using normalized ΔCt values, which constituted the primary inferential analysis. Fold-change values calculated using the 2^−ΔΔCt^ method were used for descriptive presentation and were not subjected to a separate inferential statistical analysis. Results were considered statistically significant at *p* < 0.05. Exact *p* values were reported whenever possible; values below 0.001 were reported as *p* < 0.001. All statistical analyses were performed using Statistica software (version 14; StatSoft Inc., Tulsa, OK, USA).

## 5. Conclusions

Overall, the above findings indicate that biological therapies targeting IL-6 and TNF-α pathways exerted the most pronounced effects on systemic inflammatory and neurotrophic parameters in the CIA model. Infliximab was associated with reduced IL-6 concentrations and a lower IL-6/IL-10 ratio, whereas tocilizumab was associated with increased IL-10 and elevated circulating BDNF levels. These changes suggest that targeted cytokine inhibition may shift the systemic immune profile toward a more anti-inflammatory state and may be linked to neurotrophic regulation. In contrast, methotrexate and sertraline produced more moderate or selective effects, while untreated CIA was characterized by sustained immune activation, particularly reflected by elevated IL-15 levels. MSTN and irisin remained largely unchanged, suggesting that the applied interventions did not induce consistent systemic changes in these myokines. Similarly, central transcriptional changes were limited, with a significant overall difference observed for striatal BDNF expression but no significant between-group differences in GFAP or IL-1β expression. Together, these results highlight the differential impact of therapeutic strategies on immune–neurotrophic interactions in CIA and suggest that biological cytokine-targeted therapies may influence systemic inflammatory balance more strongly than conventional treatment approaches.

## Figures and Tables

**Table 1:** Circulating concentrations of inflammatory, neurotrophic, and muscle-derived biomarkers in experimental groups.

Experimental Group (Treatment)	IL-6 (pg/mL)	IL-10 (pg/mL)	IL-6/IL-10	IL-15 (pg/mL)	BDNF (pg/mL)	MSTN (pg/mL)	Irisin (ng/mL)
A (Adjuvant control)	96.12 ± 9.79	29.72 ± 37.65	4.58 ± 2.36	75.98 ± 55.40	1262.50 ± 388.91	597.67 ± 126.87	225.45 ± 155.27
B (CIA)	103.32 ± 10.71	35.25 ± 15.38	3.29 ± 1.46	307.83 ± 13.46	1312.50 ± 106.07	605.83 ± 8.49	245.01 ± 79.21
C (CIA + MTX)	101.05 ± 9.64	59.41 ± 32.88	1.91 ± 0.68	144.40 ± 53.60	793.75 ± 742.46	437.37 ± 146.14	188.47 ± 19.74
D (CIA + SRT)	101.99 ± 10.71	53.78 ± 32.53	2.29 ± 1.41	186.66 ± 150.55	1031.25 ± 388.91	631.42 ± 62.23	255.12 ± 107.04
E (CIA + MTX + SRT)	106.92 ± 11.79	55.81 ± 12.20	1.96 ± 0.75	40.85 ± 23.49	881.25 ± 954.59	595.67 ± 154.62	223.76 ± 3.97
F (CIA + IFX)	81.73 ± 12.86	56.72 ± 23.33	1.56 ± 0.53	49.28 ± 9.58	2068.75 ± 565.69	493.83 ± 75.42	310.30 ± 335.44
G (CIA + TCZ)	107.48 ± 0.00	66.66 ± 23.33	1.65 ± 0.30	72.35 ± 6.84	3181.25 ± 989.95	558.83 ± 378.07	120.11 ± 87.74

Abbreviations: CIA—collagen-induced arthritis; MTX—methotrexate; SRT—sertraline; IFX—infliximab; TCZ—tocilizumab.

**Table 2:** The characteristics of the ELISA kits used.

Specificity			Precision		Sensitivity	Supplier	
Factor	Unit	Detection Range	Intra-Assay Variation (%)	Inter-Assay Variation (%)	Minimum Detectable Concentration	Supplier	Catalog No
IL-6	pg/mL	1000–15.6	<4.5%	<6.9%	<7.84	EIAab Science Co., Ltd., Wuhan, China	E0079r
IL-15	pg/mL	1000–15.6	<6.4%	<9.1%	<7.84	EIAab Science Co., Ltd., Wuhan, China	E0061r
IL-10	pg/mL	2000–31.2	<4.6%	<8.3%	<15.67	EIAab Science Co., Ltd., Wuhan, China	E0056r
BDNF	pg/mL	3000–12.29	<10%	<12%	<12	Invitrogen, Thermo Fisher Scientific, Waltham, MA, USA	ERBDNF
MSTN	pg/mL	2000–31.25	<8%	<10%	<18.75	FineTest, Wuhan Fine Biotech Co., Ltd., Wuhan, China	ER0573
Irisin	ng/mL	1000–0.1	<10%	<15%	=1.91	Phoenix Pharmaceuticals, Inc., Burlingame, CA, USA	EK-067-29

**Table 3:** Primer sequences, primer concentrations, and relevant references.

Target Gene	Primer Sequence (5′–3′)	Primer Concentration	References
BDNF	AGCCTCCTCTGCTCTTTCTG	1.8 µL	[63]
	CGCCGAACCCTCATAGACAT	1.8 µL	
GFAP	GGAGTGGTATCGGTCTAAGTTTGC	0.6 µL	[64]
	GTTGGCGGCGATAGTCGTTAG	1.8 µL	
IL-1β	CACCTCTCAAGCAGAGCACAG	1.8 µL	[65]
	GGGTTCCATGGTGAAGTCAAC	1.8 µL	
GAPDH	CAACTCCCTCAAGATTGTCAGCAA	1.8 µL	[66]
	GGCATGGACTGTGGTCATGA	0.6 µL	

## Data Availability

The original contributions presented in this study are included in the article. Further inquiries can be directed to the corresponding authors.

## Abbreviations

The following abbreviations are used in this manuscript:

RA	Rheumatoid arthritis
IL-1β	Interleukin-1β
IL-6	Interleukin-6
TNF-α	Tumour necrosis factor-α
IL-15	Interleukin-15
BDNF	Brain-derived neurotrophic factor
GFAP	Glial fibrillary acidic protein
SSRIs	Selective serotonin reuptake inhibitors
DMARDs	Disease-modifying antirheumatic drugs
MSTN	Myostatin
IL-10	Interleukin-10
CIA	Collagen-induced arthritis
